# Two New Compounds from Allii Macrostemonis Bulbus and Their In Vitro Antioxidant Activities

**DOI:** 10.3390/molecules28176176

**Published:** 2023-08-22

**Authors:** Jianfa Wu, Lei Li, Chang Liu, Chunyi Li, Ying Cui, Weixing Ding, Jing Zhang, Leiling Shi

**Affiliations:** 1 Department of Traditional Chinese Medicine, College of Traditional Chinese Medicinal Materials, Jilin Agricultural University, Changchun 130118, China; 20210971@mails.jlau.edu.cn (J.W.); 20201629@mails.jlau.edu.cn (L.L.); 20210969@mails.jlau.edu.cn (C.L.); 20221569@mails.jlau.edu.cn (C.L.); 20210970@mails.jlau.edu.cn (Y.C.); 20221576@mails.jlau.edu.cn (W.D.); 2 Xinjiang Institute of Chinese and Ethnic Medicine, Urumqi 830002, China

**Keywords:** Allii Macrostemonis Bulbus, biphenyl glycoside, steroidal saponin, antioxidant

## Abstract

Two new compounds named 4,4′-bis(*β*-D-glucopyranosyloxy)biphenyl (**1**) and spirostane-25(27)-en-2*α*,3*β*-diol-3-*O*-*β*-D-xylopyranosyl(1→3)-*β*-D-glucopyranosyl(1→4)-*β*-D-galactopyranoside (**2**) were isolated from n-butanol extraction part of 80% ethanol extract of Allii Macrostemonis Bulbus. Alongside these, ten known compounds (**3–12**) were also identified, including a flavonoid glycoside (**3**), seven steroids (**4**–**10**), a nucleoside (**11**), and a phenylpropanoid glycoside (**12**) were found. Notably, compounds **3**–**6** were isolated from this plant for the first time. The structures of all compounds were confirmed using high-resolution electrospray ionization mass spectrometry (HR-ESI-MS), 1D, and 2D NMR spectroscopy. Some of these compounds showed strong antioxidant activity, and compound **1** demonstrated the most potent reduction of ferric ions (Fe^3+^) with an IC_50_ value of 0.59 ± 0.18 mg/mL. Compounds **2** and **3** exhibited the highest scavenging activity against superoxide anion radicals (O_2_^−^·) with an IC_50_ value of 0.02 ± 0.01 mg/mL. Additionally, compound **3** displayed substantial scavenging activity against 2,2-diphenyl-1-picrylhydrazyl (DPPH) and 2,2′-azino-bis(3-ethylbenzothiazoline-6-sulfonic acid) (ABTS) with IC_50_ values of 0.21 ± 0.17 mg/mL and 0.02 ± 0.01 mg/mL, respectively. The discovery of these two new compounds is a reference for identifying Allii Macrostemonis Bulbus quality markers. Moreover, their exceptional antioxidant activity offers a promising avenue for uncovering novel natural antioxidants.

## 1. Introduction

**Figure 1:**
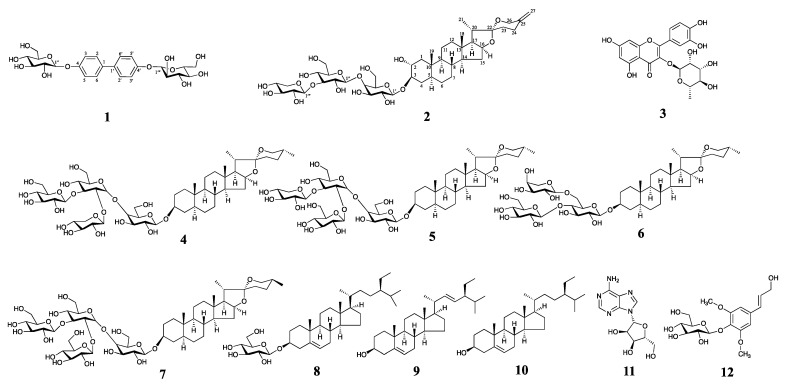
Structures of compounds **1**–**12**, compounds **1**–**2** are new compounds, and **3**–**12** are known ones.

Allii Macrostemonis Bulbus (AMB) is the dried bulb of *Allium macrostemon* Bunge or *Allium chinense* G. Don from the genus *Allium*, family Liliaceae, known as “Xiebai” in China [1]. Research on the chemical composition of AMB has never ceased. AMB contains steroidal saponins, sulfur compounds and alkaloid constituents [2]. The theory of “medicine and food coming from the same source” has a long history, and more and more attention has been paid to preventing and treating diseases with natural medicines derived from the same source of medicine and food. As a typical dual-use plant, AMB is an important traditional Chinese medicine for the treatment of “chest paralysis and cardiac pain” it often is used to treat coronary heart disease, myocardial ischemia, angina pectoris, abdominal pain and diarrhea, and hyperlipidemia [3]. In addition, AMB also has a variety of pharmacological activities, such as antioxidant [4,5,6], anti-tumor [7,8,9], lipid-lowering [10], and inhibition of platelet aggregation [11,12,13]. However, unfortunately, the quality markers of AMB have not been elucidated, which undoubtedly poses a challenge to the quality control of the herb. Oxidative stress results from an imbalance between intracellular reactive oxygen species (ROS) production and the antioxidant effects, ultimately leading to damage within the body’s biological systems when the organism faces various risk factors [14]. ROS are central to generating oxidative stress damage and primarily consist of O_2_^−^·, hydrogen peroxide, and hydroxyl radicals. Low-to-moderate levels of ROS play crucial roles in normal cellular and mitochondrial signaling and function. However, excessive ROS contribute to oxidative damage in cells and tissues, triggering various diseases [15]. Discovering safe and effective natural antioxidants is of paramount importance. Many herbal medicines contain components with scavenging ROS. Some components by binding to ROS, reduce the overall ROS levels in the body. Or they enhance antioxidant enzymes such as superoxide dismutase, glutathione peroxidase enzyme, and catalase while also decreasing the production of malondialdehyde, thereby reducing oxidative stress damage [16,17]. Therefore, we studied the n-butanol part of 80% ethanolic extract of AMB and isolated and characterized 12 compounds (Figure 1), of which **1** and **2** were new, **3**–**12** were known compounds and **3**–**6** were isolated from the plant for the first time. The structural characterization of the isolated compounds was determined using comprehensive spectral data analysis. In addition, the in vitro antioxidant activity of the isolated compounds was investigated. The structural elucidation of the isolated compounds and their potential antioxidant effects are presented.

## 2. Results and Discussion

### 2.1. Structure Elucidation

**Figure 2:**
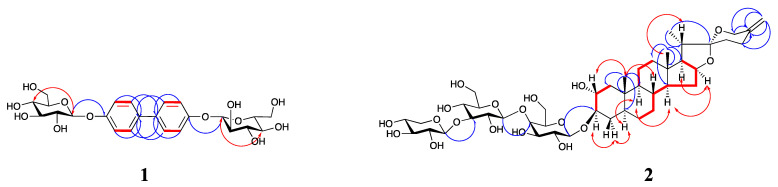
Key ^1^H-^1^H COSY (in red bold), HMBC (blue arrows) and NOESY (red arrows) correlations of compounds **1**–**2**.

Compound **1** is a yellowish-green amorphous powder with a positive Molish reaction. The *m*/*z* value of the positive ion HR-ESI-MS was 533.1657 [M + Na]^+^ (calculated 533.1635, C_24_H_30_O_12_Na^+^, Figure S1), and the combination of its ^1^H and ^13^C spectra determined its molecular formula to be C_24_H_30_O_12_ with an unsaturation degree of 10, so it was hypothesized that two benzene rings and two sugar molecules existed in the structure of compound **1**. The low-field region of ^1^H-NMR (600 MHz, Methanol-*d*_4_) of compound **1** (Table 1 and Figure S2) suggested the presence of two alkenyl hydrogens proton signals *δ_H_* 7.91 (4H, d, *J* = 8.76 Hz, H-3, 5, 3′, 5′), 6.76 (4H, d, *J* = 8.82 Hz, H-2, 6, 2′, 6′) and one sugar-terminated proton signal *δ_H_* 5.32 (2H, d, *J* = 7.50 Hz, H-1″, 1‴). The ^13^C-NMR (150 MHz, Methanol-*d*_4_) of compound **1** (Table 1 and Figure S3) suggested the presence of 10 carbon signals, of which four carbon signals were assigned to the aglycone and six carbon signals to the sugar molecule, which, combined with the above analysis, suggests that the compound is a symmetric structure and the aglycone partially biphenyl, which validates our previous speculation. The type of sugar molecule was determined by the experiment of acid hydrolysis, and the result showed that only glucose was present in compound **1**, and all of them were *β*-configuration according to their coupling constants. According to the HMQC and HMBC spectra (Figure 2, Figures S4 and S5), the proton signals of the sugar end-groups *δ_H_* 5.32 (2H, d, *J* = 7.50 Hz, H-1″, 1‴) were correlated with *δ_C_* 101.58. So it was attributed to the C-1″, 1‴ and remote correlation exists with *δ_C_* 133.42, so it is attributed to C-4, 4′. Based on the HMBC spectra *δ_H_* 7.91 (4H, d, *J* = 8.76 Hz, H-3, 5, 3′, 5′) and *δ_H_* 6.76 (4H, d, *J* = 8.82 Hz, H-2, 6, 2′, 6′) both remotely correlate with *δ_C_* 157.42, which was attributed to C-1, 1′, and then summing up with the HMQC, ^1^H-^1^H COSY and NOESY spectra (Figure 2, Figures S4, S6 and S7), we attributed *δ_C_* 115.57 and *δ_C_* 131.21 to C-2, 6, 2′, 6′ and C-3, 5, 3′, 5′, respectively. Compound **1** differs from the structure of the known compound 4,4′-bis-(*α*-D-mannopyranosyloxy)biphenyl [18] reported in the literature only by the type of sugar molecule attached to it, and therefore compound **1** was named 4,4′-bis-(*β*-D-glucopyranosyloxy)biphenyl.

Compound **2** was an amorphous white powder. Molish was positive for reaction with Liebermann-Burchard, but negative for reaction with dimethylaminobenzaldehyde hydrochloride, and showed purple-red color after heating with 10% concentrated H_2_SO_4_-EtOH on a thin silica gel plate and finally showed yellow-green color after sitting for some time, suggesting that the compound might be a spirostanol type saponin. Positive ion HR-ESI-MS gave its *m*/*z* as: 909.4487 [M + Na]^+^ (calculated 909.4460, C_44_H_70_O_18_Na^+^, Figure S8), which, combined with its ^1^H and ^13^C spectra, identified its molecular formula as C_44_H_70_O_18_. ^1^H-NMR (600 MHz, Pyridine-*d*_5_) data of compound **2** (Table 2 and Figure S9) suggested the presence of two characteristic signals of tertiary methyl protons in the high field region. at *δ_H_* 1.10 (3H, s, Me-18), 1.00 (3H, s, Me-19), and a characteristic signal for a secondary methyl proton at *δ_H_* 1.42 (3H, d, *J* = 6.42 Hz, Me-21). The ^13^C-NMR (150 MHz, Pyridine-*d*_5_) data of compound **2** (Table 2 and Figure S10) suggested the presence of 44 carbon signals, of which 27 carbon signals were assigned to saponin elements, and 17 carbon signals were assigned to the three sugar molecules. We found the presence of three methyl signals at *δ_C_* 17.00, 23.61, and 14.85, which we assigned to C-18, C-19, and C-21, respectively, based on a previous study [19], while we found that the methyl signal of C-27 was missing from the parent nucleus of the spirosterane saponin element, and the two terminal alkene hydrogen proton signals at *δ_H_* 4.80 (1H, s) and 4.83 (1H. s) were found and both were remotely correlated with *δ_C_* 145.03 (C-25), 65.50 (C-26) and 32.34 (C-24), respectively (HMBC, Figure 2 and Figure S12), thus determining the presence of a double bond at C_25_–C_27_. the proton signal of Me-21 *δ_H_* 1.42 (3H, d, *J* = 6.42 Hz) was correlated with *δ_C_* 63.46, 41.03, and 110.17, which are remotely correlated to C-17, C-20, and C-22, respectively; the proton signal *δ_H_* 1.10 (3H, s) of Me-18 is remotely correlated to *δ_C_* 40.49, 43.31, 55.71 and 63.46, which are attributed to C-12, C-13, C-14, and C-17, respectively; the proton signal of Me-19 *δ_H_* 1.00 (3H, s) is remotely correlated with *δ_C_* 39.25, 39.81, 36.53, and 37.24, which are assigned to C-1, C-5, C-9, and C-10, respectively. since the hydrogen signal of Me-18 (*δ_H_* 1.10) is in a lower field than that of Me-19 (*δ_H_* 1.00), H-5 is determined to be in the *α*-conformation [20]. In the ^1^H-^1^H COSY spectrum (Figure 2 and Figure S13), there is a correlation between H_2_-1/H-2/H-3, and combined with the HMQC spectrum (Figure S11), so *δ_C_* 67.45 is attributed to C-2 and *δ_C_* 82.27 is attributed to the signal at C-3 position; since *δ_C_* 67.45 belongs to for the hypomethyl carbon signals attached to oxygen atoms, suggesting that there may be a hydroxyl group attached to the C-2 position, meanwhile, considering that C-3 is affected by the glycosylation shift resulting in a chemical shift to the lower field of 82.28 ppm, so it is presumed that the C-3 position is attached to the sugar group. The hydrogen signal at the C-2 position can be seen in the NOESY spectrum (Figure 2 and Figure S14) associated with the Me-19 (*β*-conformation) signal, thus identifying the hydroxyl group at the C-2 position as *α*-conformation [21].

The type of sugar molecules was determined experimentally by acid hydrolysis, which showed a 1:1:1 ratio of galactose, glucose, and xylose. Based on the three isomeric proton signals *δ_H_* 4.94 (1H, d, *J* = 7.62 Hz, H-1′), 5.31 (1H, d, *J* = 7.68 Hz, H-1″) and 5.34 (1H, d, *J* = 7.74 Hz, H-1‴) inferred the presence of three sugar molecules, and the correlations of HMQC (Figure S11) with the three isomeric carbons were 103.08, 106.62, and 107.55 ppm, respectively, verifying this conjecture. All of them could be judged as *β*-conformation based on the coupling constants of the three. In the HMBC spectrum (Figure 2 and Figure S12), the anomeric proton signal *δ_H_* 4.94 (1H, d, *J* = 7.62 Hz) of galactose was remotely correlated with the parent nucleus C-3 (*δ_C_* 82.27), so it was judged that the sugar was attached to the C-3 position of the parent nucleus of saponin meta, which was consistent with our previous judgment. The anomeric proton signal *δ_H_* 5.31 (1H, d, *J* = 7.68 Hz) of glucose is remotely correlated with C-4 (*δ_C_* 70.33) of galactose, and the linkage is presumed to be 1→4. The anomeric proton signal *δ_H_* 5.34 (1H, d, *J* = 7.74 Hz) of xylose is remotely correlated with C-3 (*δ_C_* 79.82) of glucose, and the linkage is presumed to be 1→3. In summary, the structure of compound **2** was identified as spirostane-25(27)-ene-2*α*,3*β*-diol-3-*O*-*β*-D-xylopyranosyl (1→3)-*β*-D-glucopyranosyl (1→4)-*β*-D-galactopyranoside.

In addition, the structures of quercitrin (**3**) [22], dongnoside E (**4**) [23], desgalactotigonin (**5**) [24], smilaxin C (**6**) [25], macrostemonoside A (**7**) [26], daucosterol (**8**) [27], stigmasterol (**9**) [28], *β*-sitosterol (**10**) [29], adenosine (**11**) [30] and syringin (**12**) [31], were determined by comparison with spectral data reported in the literature (Figure 1, see Table S1 for carbon spectral data). Among them, compounds **3**–**6** were first discovered from the plant.

### 2.2. Antioxidant Activity

Using VC as a positive control, we evaluated the in vitro antioxidant activities of compounds **1**–**12**. These evaluations included scavenging capacity assays for DPPH, ABTS, and O_2_^−^· radicals and reducing capacity assays for Fe^3+^. As shown in Table 3, the results indicate that all compounds exhibited strong scavenging ability against ABTS radicals. Among them, compounds **1** and **2** demonstrated favorable activity in scavenging other radicals and reducing Fe^3+^. This may be related to the sugar groups and double bonds to which they are attached. Moreover, compound **3** displayed the most potent scavenging ability among all three types of radicals, likely attributed to the presence of phenolic hydroxyl groups in its structure. Interestingly, we found that these compounds had relatively poor scavenging activity against DPPH radicals and generally strong scavenging activity against ABTS radicals, which may be related to the different modes of production and scavenging mechanisms of the two radicals, suggesting that a single in vitro antioxidant assay is not capable of elucidating the antioxidant activities of the compounds definitively.

## 3. Materials and Methods

### 3.1. General Experimental Procedures

NMR spectra were recorded on a Bruker Avance III 600 spectrometer (Bruker, Billerica, Germany) with ^1^H-NMR at 600 MHz and ^13^C-NMR at 150 MHz, with chemical shift values expressed as *δ* values, using deuterated solvent signals as an internal reference. HR-ESI-MS was performed using an LTQ-Orbitrap XL spectrometer (Thermo Fisher Scientific, Boston, MA, USA). Acchrom S6000 high-performance liquid chromatograph (Acchrom Tech Technology Co., Ltd., Beijing, China) was used for analysis with an ELSD-UM 5800 Plus (Unimicro Technologies Co., Ltd., Shanghai, China). Column chromatography (CC) analysis was performed using silica gel (200–300 mesh and 300–400 mesh, Qingdao Ocean Chemical Factory, Qingdao, China) and C_18_ reverse silica gel packing (50 μm, YMC Co., Ltd., Tokyo, Japan). Analytically pure solvents (petroleum ether, ethyl acetate, ethanol, methanol, n-BuOH, and dichloromethane) (Beijing Chemical Factory, Beijing, China) were used for extraction and CC separation. Chromatographically pure methanol (Thermo Fisher Scientific, Waltham, MA, USA) was used for high-performance liquid phase analysis. Deuterated solvents (Deuterated methanol, deuterated pyridine, deuterated chloroform, and deuterated dimethyl sulfoxide) (Aladdin Biochemical Technology Co., Ltd., Shanghai, China) were used for nuclear magnetic resonance spectroscopy.

### 3.2. Plant Material

The experimental plants were purchased from Changchun Chinese Herb Shop (Changchun, China) in 2021. It was identified as *Allium macrostemon* Bunge from the genus *Allium*, family Liliaceae by Prof. Jing Zhang of the College of Traditional Chinese Medicine, Jilin Agricultural University, and the voucher specimen (20210971) was deposited in the Herbal Library of the College of Traditional Chinese Medicine, Jilin Agricultural University.

### 3.3. Isolation and Purification of Compounds ***1***–***12***

The AMB sample (3.0 kg) was pulverized, passed through a 40-mesh sieve, and dried to obtain AMB powder (2.76 kg). AMB powder (2.76 kg) was extracted by adding 80% EtOH solution in the ratio of 1:5 (m:V, g:mL) for 1 h, and then extracted for 30 min with the help of 100 W ultrasonic waves, filtered, and repeated for six times. Then the filtrate was combined and concentrated under reduced pressure until it was free of alcohol flavor and then lyophilized to obtain AMB ethanol extract (1401 g). 500 g of AMB ethanol extract was dissolved in water (m:V = 1:15, g:mL) and then extracted sequentially with petroleum ether, CH_2_Cl_2_, and n-BuOH in a gradient. Each solvent was extracted four times, the petroleum ether and CH_2_Cl_2_ portions were discarded to remove the fat-soluble and less polar components, and the n-BuOH portion was concentrated under reduced pressure to be free of alcohol and then lyophilized to obtain the n-BuOH extract part of AMB (11.3 g). 5 g of n-BuOH extract fraction of AMB was subjected to CC analysis on 200–300 mesh silica gel, eluted with a gradient of CH_2_Cl_2_-MeOH-H_2_O (5:2:1~13:8:2) to obtain 12 fractions (Fr. 1~12). Fr. 2 (940 mg) was subjected to CC on 300–400 mesh silica gel, eluted with a gradient of CH_2_Cl_2_-EtOAc (20:1~1:1) to give four subfractions (Fr. 2.1~2.4), Fr. 2.1 (51 mg) was recrystallized in MeOH to give compound **8** (10 mg), Fr. 2.3 (72 mg) and Fr. 2.4 (83 mg) were subjected to CC on ODS silica gel (50 μm) eluted with MeOH-H_2_O (10–30%) gradient to give compound **9** (54 mg) and compound **10** (56 mg), respectively; Fr. 3 (185 mg) was subjected to CC on ODS silica gel (50 μm) eluted with MeOH-H_2_O (10–50%) gradient elution to give three subfractions (Fr. 3.1~3.3), Fr. 3.2 (81 mg) was subjected to CC on ODS silica gel (50 μm), eluted with MeOH-H_2_O (20–40%) gradient to give Compound **12** (22 mg), Compound **6** (23 mg), and Compound **7** (26 mg); Fr. 5 (194 mg) was subjected to CC on ODS silica gel (50 μm), eluted with MeOH-H_2_O (20–70%) gradient to afford three subfractions (Fr. 5.1~5.3), Fr. 5.1 (25 mg) and Fr. 5.2 (33 mg) were recrystallized in MeOH to afford compound **4** (21 mg) and compound **5** (27 mg), respectively; Fr. 6 (77 mg) was recrystallized in MeOH to give compound **11** (56 mg); and Fr. 7 (58 mg) was subjected to CC on ODS silica gel (50 μm) and eluted with a gradient of MeOH-H_2_O (20–70%) to give compound **1** (11 mg) and compound **3** (7 mg).

### 3.4. Antioxidant Activity

#### 3.4.1. Preparation of Sample Solutions

Compounds **1**–**12** and positive control drug vitamin C (VC) were weighed 6.0 mg each, add methanol, ultrasound-assisted dissolution was configured into 1 mg·mL^−1^ master batch, and each master batch was gradient diluted into 0.5 mg·mL^−1^, 0.25 mg·mL^−1^, 0.125 mg·mL^−1^, 0.0625 mg·mL^−1^ of sample solution, respectively.

#### 3.4.2. Measurement of DPPH Free Radical Scavenging Capacity

The method of reference [32] was slightly modified. 100 μL of sample solution was accurately pipetted into a 96-well plate. Then 100 μL of DPPH solution (0.2 M) was added, and the wells were blown up uniformly; then the reaction was performed in a thermostat at 25 °C for 30 min under light protection. After the reaction, the absorbance value (A_x_) was measured at 517 nm. In the control group, anhydrous ethanol replaced the sample solution (A_0_) and DPPH solution (A_y_), respectively. VC was used as a positive control, and the procedure was the same as above. All measurements were set up with three replicate wells in parallel, and the clearance was calculated according to Equation (1).

#### 3.4.3. Measurement of ABTS Radical Scavenging Capacity

The method of reference [33] was slightly modified. 19.2 mg of ABTS and 3.31 mg of potassium persulfate were weighed precisely, dissolved with water, and then fixed to a 5 mL volumetric flask to obtain the mother liquor of ABTS. After 14 h of reaction at room temperature and protected from light, the mother liquor was diluted with anhydrous ethanol until the absorbance at 734 nm was 0.7 ± 0.1, which was obtained as ABTS solution. After 100 μL of sample solution was accurately aspirated into a 96-well plate, 100 μL of ABTS solution was added. Each well was blown up uniformly and then reacted in a thermostat at 25 °C for 30 min under light protection, and the absorbance value (A_x_) was measured at 734 nm after the reaction was completed. In the control group, anhydrous ethanol replaced the sample solution (A_0_) and ABTS solution (A_y_). VC was used as a positive control, and the procedure was the same as above. All measurements were set up with three replicate wells in parallel, and the clearance was calculated according to Equation (1).

#### 3.4.4. Measurement of O_2_^−^· Scavenging Capacity

The method of reference [34] was slightly modified. Accurately aspirate 90 μL Tris-HCl solution (50 mM, pH 8.2) in a 96-well plate, let it stand for 30 min at 25 °C in a thermostat, then add 50 μL of the sample solution, and quickly add eight μL of pyrogallic gallic acid solution (25 mM). The reaction was carried out for 5 min at 25 °C in a thermostat. Then 20 μL of HCl solution (10 M) was added to terminate the reaction, and the absorbance values (A_x_) were measured at 325 nm. In the control group, HCl solution (10 M) was used instead of pyrogallic gallic acid solution (A_y_), and distilled water was used instead of sample solution (A_0_), respectively. VC was used as a positive control, and the procedure was the same as above. All measurements were set up with three replicate wells in parallel, and the clearance was calculated according to Equation (1).

#### 3.4.5. Measurement of Fe^3+^ Reduction Capacity

The method of reference [35] was slightly modified. Precisely 200 μL of sample solution was aspirated into a 2 mL centrifuge tube, and phosphate buffer solution (pH 6.6) and 200 μL of 1% potassium hexacyanoferrate solution were added sequentially. After mixing, the reaction was carried out in a thermostat at 50 °C for 30 min. The reaction was terminated by adding 200 μL of 10% trichloroacetic acid solution after cooling at room temperature and then centrifuged at 3500 r/m for 15 min. 400 μL of the supernatant was drawn, and 400 μL of distilled water was added. This was followed by adding 80 μL of 0.1% ferric chloride solution. The mixture was then thoroughly mixed, and 200 μL of the supernatant was drawn into a 96-well plate. Absorbance values (A_x_) were measured at 700 nm. In the control group, distilled water was used instead of the sample solution (A_0_). VC was used as a positive control, and the procedure was the same as above. All measurements were set up with three replicate wells in parallel, and the clearance was calculated according to Equation (2).

DPPH, ABTS or O_2_^−^· radical scavenging rate (%) = [A_0_ − (A_x_ − A_y_)]/A_0_ × 100% (1)

Fe^3+^ reduction rate (%) = (A_x_ − A_0_) × 100% (2)

#### 3.4.6. Statistical Analysis

IC_50_ values (concentration of test sample required to scavenge 50% of free radicals or reduce 50% of Fe^3+^) were determined by non-linear regression using GraphPad Prism software and expressed as mean ± SD.

## 4. Conclusions

AMB is extensively distributed throughout the Asian region and has found widespread use in various traditional Chinese medicine formulations and dishes due to its significant medicinal and nutritional value. Its chemical composition is intricate, contributing to its versatile efficacy. However, the investigation into its active ingredients and mechanism of action remains insufficiently explored. In the present study, twelve compounds, including two new compounds (**1**, **2**), four compounds (**3**–**6**) first identified in the AMB, and six known compounds (**7**–**12**), were isolated from the n-butanol part of 80% ethanol extract of AMB. The results of in vitro antioxidant assay showed that compounds **1** and **2** have potential biological activities, which will be elucidated in detail in our next study. The present study provides a theoretical basis for the further exploitation of AMB and the discovery of its quality markers.

## Figures and Tables

**Table 1:** The assignment of carbon and proton signals of compound **1** (in Methanol-*d*_4_).

Position	*δ_C_* (ppm)	*δ_H_* (*J* in Hz)
1, 1′	157.15	-
2, 6, 2′, 6′	115.57	6.76 (4H, d, *J* = 8.82 Hz)
3, 5, 3′, 5′	131.21	7.91 (4H, d, *J* = 8.76 Hz)
4, 4′	133.42	-
Glc-1″, 1‴	101.58	5.32 (2H, d, *J* = 7.50 Hz)
2″, 2‴	77.92	2.97 (2H, m)
3″, 3‴	76.90	3.11 (2H, m)
4″, 4‴	74.68	3.05 (2H, s)
5″, 5‴	70.27	2.30 (2H, m)
6″, 6‴	61.24	3.45 (4H, d, *J* = 11.40 Hz)

**Table 2:** The assignment of carbon and proton signals of compound **2** (in Pyridine-*d*_5_).

Position	*δ_C_* (ppm)	*δ_H_* (*J* in Hz)	Position	*δ_C_* (ppm)	*δ_H_* (*J* in Hz)
1	39.25	1.37 (1H, m) 0.94 (1H, m)	C-3 Gal-1′	103.08	4.94 (1H, d, *J* = 7.62 Hz)
2	67.45	3.83 (1H, m)	2′	82.15	4.48 (1H, m)
3	82.27	4.47 (1H, m)	3′	75.66	4.25 (1H, m)
4	29.52	1.45 (2H, m)	4′	70.33	4.55 (1H, m)
5	36.53	1.59 (1H, m)	5′	77.06	4.06 (1H, m)
6	25.30	0.90 (2H, m)	6′	62.60	4.52 (1H, m) 4.60 (1H, m)
7	26.48	1.17 (1H, m) 1.74 (1H, m)	4-Glc-1″	106.62	5.31 (1H, d, *J* = 7.68 Hz)
8	35.11	1.63 (1H, m)	2″	75.92	4.33 (1H, m)
9	39.81	1.40 (1H, m)	3″	79.82	3.83 (1H, m)
10	37.24	-	4″	72.10	4.23 (1H, m)
11	21.60	1.29 (1H, m) 2.08 (1H, m)	5″	78.45	3.81 (1H, m)
12	40.49	1.27 (1H, m) 2.04 (1H, m)	6″	63.16	4.30 (1H, m) 4.38 (1H, m)
13	43.31	-	3-Xyl-1‴	107.55	4.70 (1H, d, *J* = 7.74 Hz)
14	55.71	3.62 (1H, m)	2‴	76.11	4.35 (1H, m)
15	31.48	1.80 (1H, m) 1.82 (1H, m)	3‴	78.95	4.28 (1H, m)
16	81.69	4.69 (1H, m)	4‴	71.17	4.30 (1H, m)
17	63.46	4.43 (1H, m)	5‴	63.31	4.45 (1H, m) 4.50 (1H, m)
18	17.00	1.10 (3H, s)			
19	23.61	1.00 (3H, s)			
20	41.03	2.18 (1H, m)			
21	14.85	1.42 (3H, d, *J* = 6.42 Hz)			
22	110.17	-			
23	28.64	1.59 (1H, m) 2.21 (1H, m)			
24	32.34	1.80 (1H, m) 1.78 (1H, m)			
25	145.03	-			
26	65.50	4.46 (1H, m) 4.03 (1H, m)			
27	109.17	4.83 (1H, s) 4.80 (1H, s)			

**Table 3:** The scavenging ability of compounds **1**–**12** for DPPH, ABTS and O_2_^−^· radicals and reduction of Fe^3+^ (*n* = 3).

Compounds	IC_50_			
	DPPH	ABTS	O_2_^−^·	Fe^3+^
**1** (mg/mL)	0.68 ± 0.11	0.03 ± 0.01	0.06 ± 0.02	0.59 ± 0.18
**2** (mg/mL)	0.78 ± 0.22	0.05 ± 0.01	0.02 ± 0.01	1.37 ± 0.57
**3** (mg/mL)	0.21 ± 0.17	0.02 ± 0.01	0.02 ± 0.01	0.92 ± 0.22
**4** (mg/mL)	1.17 ± 0.12	0.26 ± 0.07	0.11 ± 0.03	1.77 ± 0.37
**5** (mg/mL)	1.18 ± 0.12	0.28 ± 0.05	0.11 ± 0.05	1.64 ± 0.30
**6** (mg/mL)	>10	0.23 ± 0.07	0.15 ± 0.05	5.35 ± 1.44
**7** (mg/mL)	0.74 ± 0.21	0.04 ± 0.02	0.27 ± 0.07	1.28 ± 0.22
**8** (mg/mL)	0.98 ± 0.18	0.09 ± 0.41	7.97 ± 0.27	3.35 ± 0.98
**9** (mg/mL)	>10	0.17 ± 0.07	2.83 ± 0.17	>10
**10** (mg/mL)	>10	0.16 ± 0.04	0.45 ± 0.21	>10
**11** (mg/mL)	1.47 ± 0.24	0.23 ± 0.05	7.50 ± 1.22	2.11 ± 0.09
**12** (mg/mL)	>10	0.11 ± 3.48	0.04 ± 0.02	1.37 ± 0.57
VC (μg/mL)	1.94 ± 0.11	1.23 ± 0.14	72.86 ± 3.32	85.45 ± 4.65

IC_50_ values of compounds **1**–**12** are in mg/mL, and IC_50_ values of VC are in μg/mL. IC_50_ values are expressed as means ± SD.

## Data Availability

The data presented in this study are available in the Supplementary Materials.

## Supplementary Materials

The following supporting information can be downloaded at: https://www.mdpi.com/article/10.3390/molecules28176176/s1, Table S1: The assignment of carbon signals of compound **3**–**12**; Figure S1: HR-ESI-MS spectrum of compound **1**; Figure S2: ^1^H-NMR spectrum of compound **1** (in Methanol-*d*_4_, 600 MHz); Figure S3: ^13^C-NMR-APT spectrum of compound **1** (in Methanol-*d*_4_, 150 MHz); Figure S4: HMQC spectrum of compound **1**; Figure S5: HMBC spectrum of compound **1**; Figure S6: COSY spectrum of compound **1**; Figure S7: NOESY spectrum of compound **1**; Figure S8: HR-ESI-MS spectrum of compound **2**; Figure S9: ^1^H-NMR spectrum of compound **2** (in Pyridine-*d*_5_, 600 MHz); Figure S10: ^13^C-NMR-APT spectrum of compound **2** (in Pyridine-*d*_5_, 150 MHz); Figure S11: HMQC spectrum of compound **2**; Figure S12: HMBC spectrum of compound **2**; Figure S13: COSY spectrum of compound **2**; Figure S14: NOESY spectrum of compound **2**.

## Sample Availability

Samples of compounds **1**–**12** are available from the authors.
