# Supplementary material for: Two New Compounds from Allii Macrostemonis Bulbus and Their In Vitro Antioxidant Activities
Source: Molecules. 2023 Aug 22;28(17):6176. doi: 10.3390/molecules28176176 (PMC10488416; doi:10.3390/molecules28176176)
Supplement: Supplementary file 1 [file molecules-28-06176-s001.zip › molecules-2560621-supplementary.pdf]

SUPPLEMENTARY MATERIAL

Two New Compounds from Allii Macrostemonis Bulbus and Their In Vitro Antioxidant Activities

Jianfa Wu [1,†], Lei Li [1,†], Chang Liu [1], Chunyi Li [1], Ying Cui [1], Weixing Ding [1], Jing Zhang [1,*] and Leiling Shi [2,*]

[1] Department of Traditional Chinese Medicine, College of Traditional Chinese Medicinal Materials, Jilin Agricultural University, Changchun 130118, China; 20210971@mails.jlau.edu.cn (J.W.); 20201629@mails.jlau.edu.cn (L.L.); 20210969@mails.jlau.edu.cn (C.L.); 20221569@mails.jlau.edu.cn (C.L.); 20210970@mails.jlau.edu.cn (Y.C.); 20221576@mails.jlau.edu.cn (W.D.)

[2] Xinjiang Institute of Chinese and Ethnic Medicine, Urumqi 830002, China

* Correspondence: zhangjing4693@jlau.edu.cn (J.Z.); shileiling@sina.com (L.S.)

† These authors contributed equally to this work.

Abstract: Two new compounds named 4,4′-bis(*β*-D-glucopyranosyloxy)biphenyl (**1**) and spirostane-25(27)-en-2*α*,3*β*-diol-3-*O*-*β*-D-xylopyranosyl(1→3)-*β*-D-glucopyranosyl(1→4)-*β*-D-galactopyranoside (**2**) were isolated from n-butanol extraction part of 80% ethanol extract of Allii Macrostemonis Bulbus. Alongside these, ten known compounds (**3–12**) were also identified, including a flavonoid glycoside (**3**), seven steroids (**4–10**), a nucleoside (**11**), and a phenylpropanoid glycoside (**12**) were found. Notably, compounds **3–6** were isolated from this plant for the first time. The structures of all compounds were confirmed using high-resolution electrospray ionization mass spectrometry (HR-ESI-MS), 1D, and 2D NMR spectroscopy. Some of these compounds showed strong antioxidant activity, and compound **1** demonstrated the most potent reduction of ferric ions (Fe^{3+}) with an IC_{50} value of 0.59 ± 0.18 mg/mL. Compounds **2** and **3** exhibited the highest scavenging activity against superoxide anion radicals ($O_2^{-}\cdot$) with an IC_{50} value of 0.02 ± 0.01 mg/mL. Additionally, compound **3** displayed substantial scavenging activity against 2,2-diphenyl-1-picrylhydrazyl (DPPH) and 2,2′-azino-bis(3-ethylbenzothiazoline-6-sulfonic acid) (ABTS) with IC_{50} values of 0.21 ± 0.17 mg/mL and 0.02 ± 0.01 mg/mL, respectively. The discovery of these two new compounds is a reference for identifying Allii Macrostemonis Bulbus quality markers. Moreover, their exceptional antioxidant activity offers a promising avenue for uncovering novel natural antioxidants.

Keywords: Allii Macrostemonis Bulbus; biphenyl glycoside; steroidal saponin; antioxidant

CONTENTS

Table S1. The assignment of carbon signals of compound **3-12** **3**

Figure S1. HR-ESI-MS spectrum of compound **1** **6**

Figure S2. ^{1}H-NMR spectrum of compound **1** (in Methanol-d_4, 600 MHz) **6**

Figure S3. ^{13}C-NMR-APT spectrum of compound **1** (in Methanol-d_4, 150 MHz) **7**

Figure S4. HMQC spectrum of compound **1** **7**

Figure S5. HMBC spectrum of compound **1** **8**

Figure S6. COSY spectrum of compound **1** **8**

Figure S7. NOESY spectrum of compound **1** **9**

Figure S8. HR-ESI-MS spectrum of compound **2** **9**

Figure S9. ^{1}H-NMR spectrum of compound **2** (in Pyridine-d_5, 600 MHz) **10**

Figure S10. ^{13}C-NMR-APT spectrum of compound **2** (in Pyridine-d_5, 150 MHz) **10**

Figure S11. HMQC spectrum of compound **2** **11**

Figure S12. HMBC spectrum of compound **2** **11**

Figure S13. COSY spectrum of compound **2** **12**

Figure S14. NOESY spectrum of compound **2** **12**

Table S1. The assignment of carbon signals of compound **3-12**

Position	δ_C(ppm)									
	**3**[a]	**4**[b]	**5**[b]	**6**[c]	**7**[b]	**8**[b]	**9**[d]	**10**[d]	**11**[b]	**12**[c]
1	-	37.61	37.41	36.54	36.54	37.95	37.37	37.37	-	133.78
2	158.54	30.35	30.15	29.04	29.05	30.73	31.77	31.78	153.77	152.74
3	136.22	78.33	78.13	76.35	75.94	79.11	71.94	71.95	-	104.42
4	179.65	35.24	35.04	34.01	34.02	39.81	42.33	42.44	150.43	132.62
5	163.24	45.04	44.85	44.04	44.04	141.36	140.87	140.87	121.96	104.42
6	99.81	29.35	29.16	28.32	28.32	122.43	121.86	121.88	158.19	152.74
7	165.95	32.83	32.63	31.89	31.89	32.65	31.77	34.04	-	128.46
8	94.70	35.66	35.46	34.66	34.66	32.51	32.02	32.01	141.04	130.21
9	159.32	54.78	54.59	53.68	53.68	50.79	50.25	50.23	91.29(Rib-C-1')	61.50
10	105.87	36.24	36.04	35.33	35.33	37.40	36.64	36.63	76.02(C-2')	102.53(Glc-C-1')
11	122.84(C-1')	21,70	21.50	20.60	20.60	21.76	21.19	19.98	72.89(C-3')	74.19(C-2')
12	116.89(C-2')	40.56	40.37	40.02	39.44	40.41	39.79	39.88	88.28(C-4')	77.27(C-3')
13	146.44(C-3')	41.21	41.01	40.12	40.12	42.95	42.42	42.42	63.50(C-5')	69.93(C-4')
14	149.82(C-4')	56.85	56.66	55.63	55.63	57.28	56.04	56.15		76.57(C-5')
15	116.36(C-5')	32.58	32.38	31.43	31.42	24.99	24.50	23.17		60.89(C-6')
16	122.94(C-6')	81.59	81.39	79.33	79.41	29.04	29.08	26.12		56.36(OCH$_3$×2)
17	103.55(Man-C-1'')	63.45	63.25	61.90	61.90	56.69	56.98	56.88		
18	72.04(C-2'')	17.08	16.88	16.25	16.25	12.45	12.18	12.12		
19	72.08(C-3'')	12.74	12.55	12.12	12.12	19.91	19.11	19.55		
20	73.22(C-4'')	42.42	42.23	40.05	41.12	36.88	40.67	36.28		
21	71.90(C-5'')	15.52	15.32	14.70	14.70	19.49	21.26	18.91		
22	17.66(C-6'')	109.69	109.49	108.42	108.42	34.66	138.48	28.39		
23		32.25	32.05	30.91	30.91	26.79	129.37	24.44		
24		29.71	29.52	28.50	28.50	46.49	51.37	45.93		
25		31.06	30.86	29.84	29.84	29.89	32.02	29.22		
26		67.30	67.11	67.57	65.92	19.67	21.36	19.55		

27	17.80	17.61	17.14	17.14	20.48	19.55	19.15
28	-		-	-	23.85	25.56	21.21
29	-		-	-	12.64	12.42	12.00
Gla-1	102.83	102.63	100.90	100.90	-		
2	75.76	75.80	73.82	71.52	-		
3	75.99	75.84	75.89	73.80	-		
4	81.97	81.36	80.21	79.28	-		
5	77.73	76.47	74.91	74.30	-		
6	61.05	60.85	68.93	59.47	-		
Glc-1	105.02	104.83	102.48	103.37	103.04		
2	80.76	81.77	74.29	80.20	75.84		
3	88.93	88.74	77.03	86.90	79.03		
4	71.29	71.82	69.96	69.62	72.14		
5	79.13	80.57	76.83	76.06	78.53		
6	62.71	62.51	63.27	60.81	63.29		
Glc-1	105.41	105.21	-	102.95			
2	76.04	77.54	-	73.54			
3	80.72	80.52	-	76.36			
4	73.68	73.48	-	69.15			
5	78.03	78.93	-	75.87			
6	63.51	63.32	-	60.97			
Glc-1	-	-	-	102.50			
2	-	-	-	73.68			
3	-	-	-	76.99			
4	-	-	-	70.11			
5	-	-	-	76.52			
6	-	-	-	61.41			
Xyl-1	105.60	105.40	-				
2	76.67	77.83	-				
3	79.17	78.98	-				

4	72.02	75.57	-
5	67.30	67.11	-
Ara-1			103.33
2			71.48
3			73.54
4			69.61
5			65.91

^{1}H-NMR and ^{13}-NMR were measured at 150 MHz and 600 MHz, respectively, using deuterated [a)] Methanol-d_4, [b)]Pyridine-d_5, [c)]DMSO-d_6, [d)]Chloroform-d as the deuterated solvent.

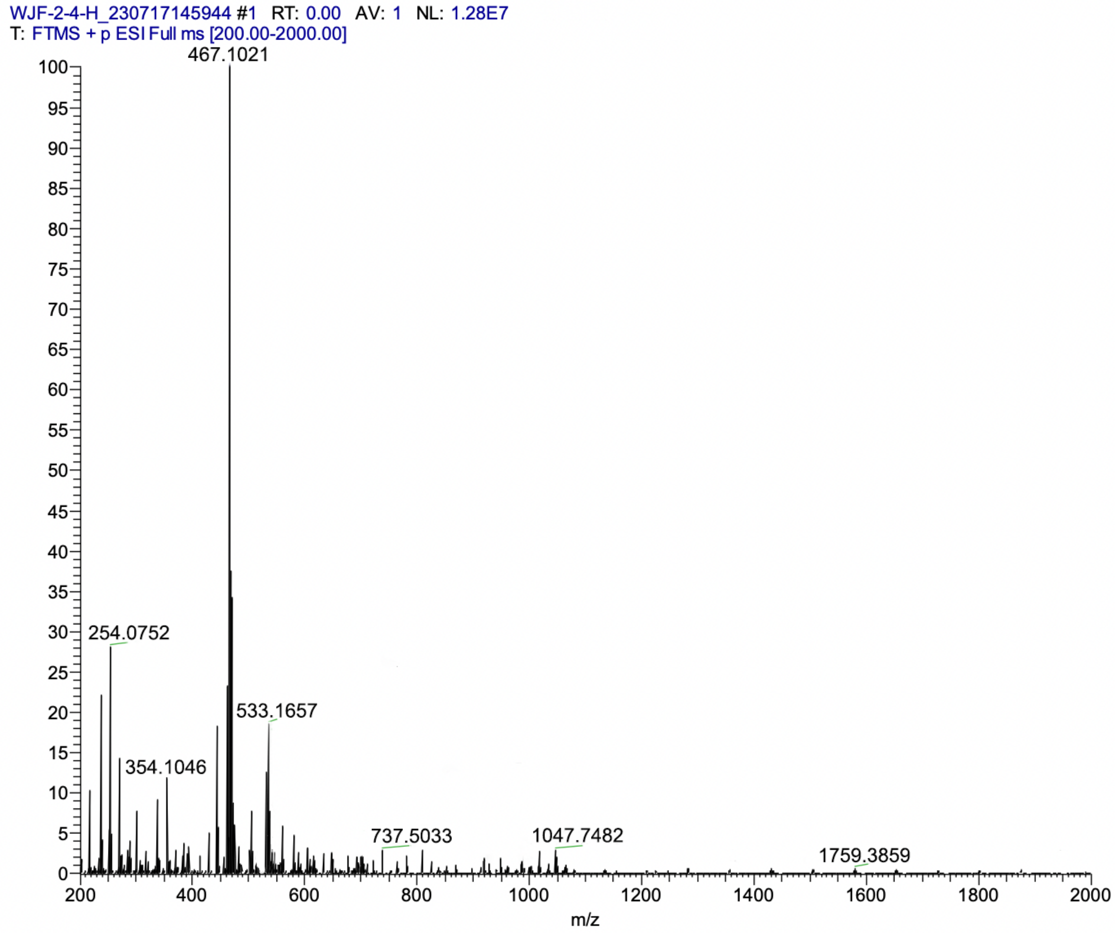

Figure S1. HR-ESI-MS spectrum of compound **1**

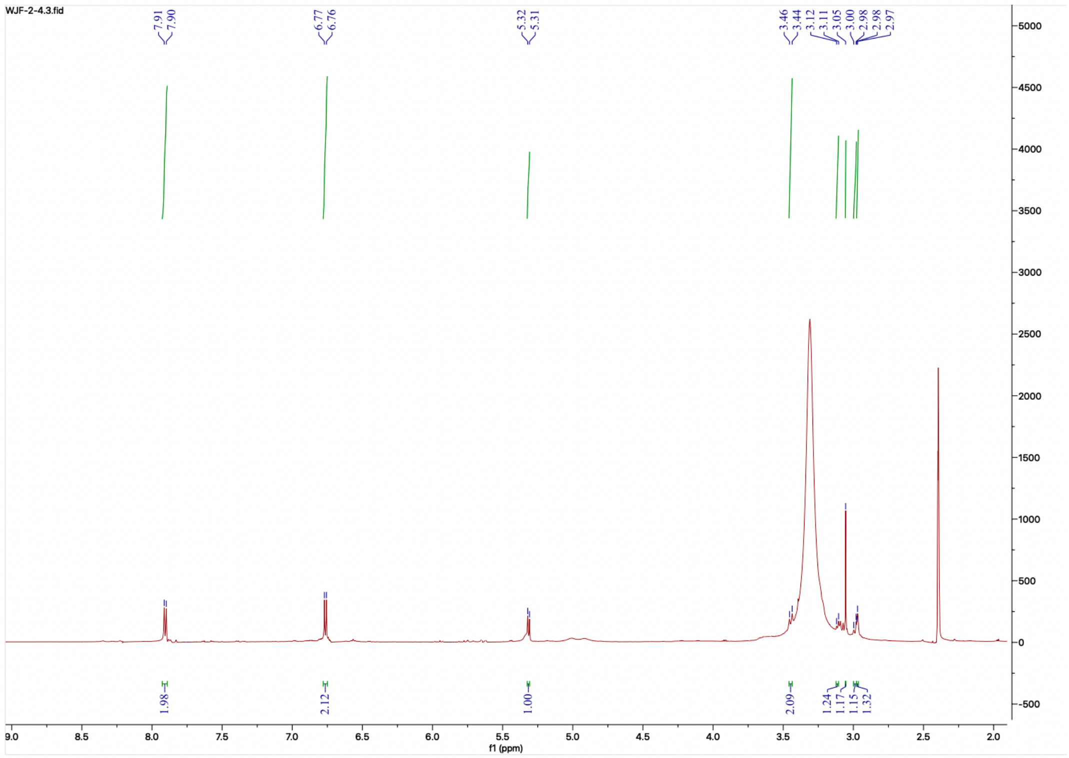

Figure S2. ^{1}H-NMR spectrum of compound **1** (in Methanol-d_4, 600 MHz)

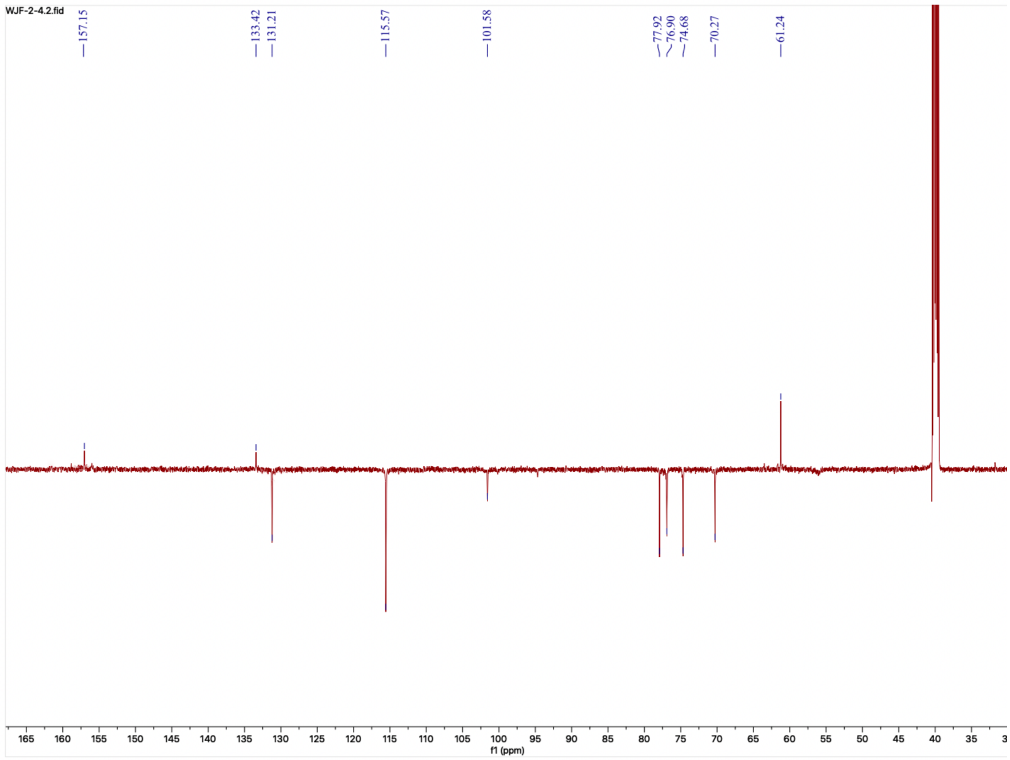

Figure S3. ^{13}C-NMR-APT spectrum of compound **1** (in Methanol-d_4, 150 MHz)

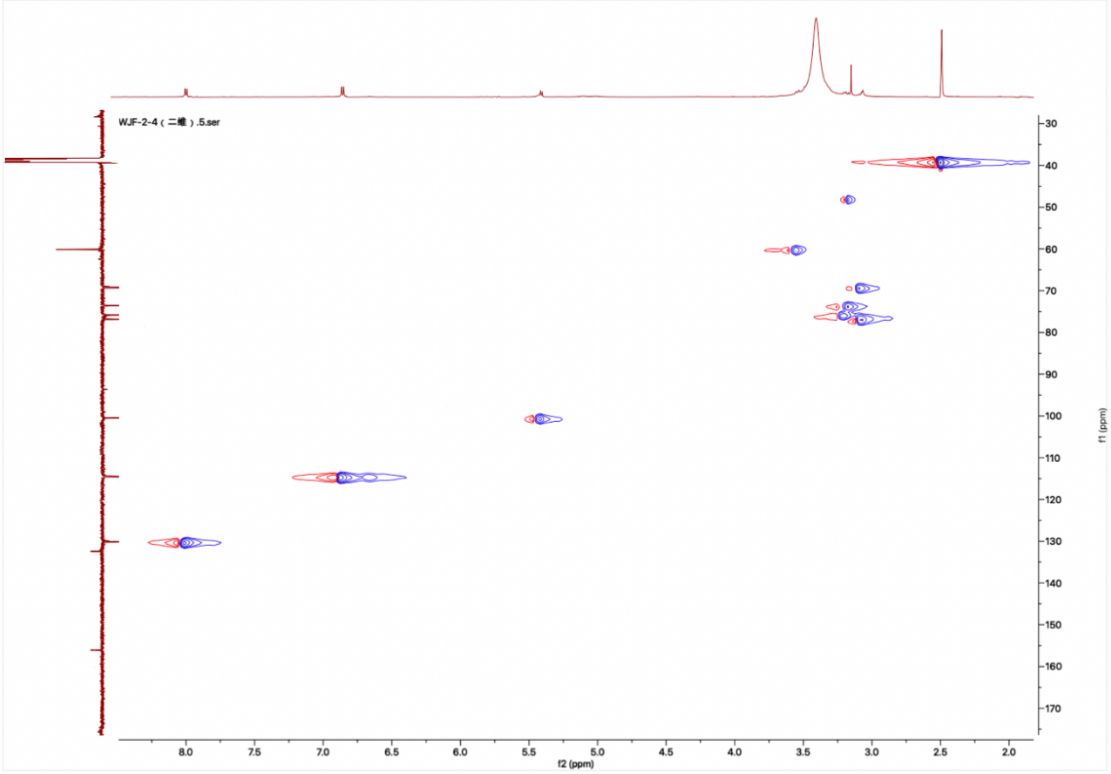

Figure S4. HMQC spectrum of compound **1**

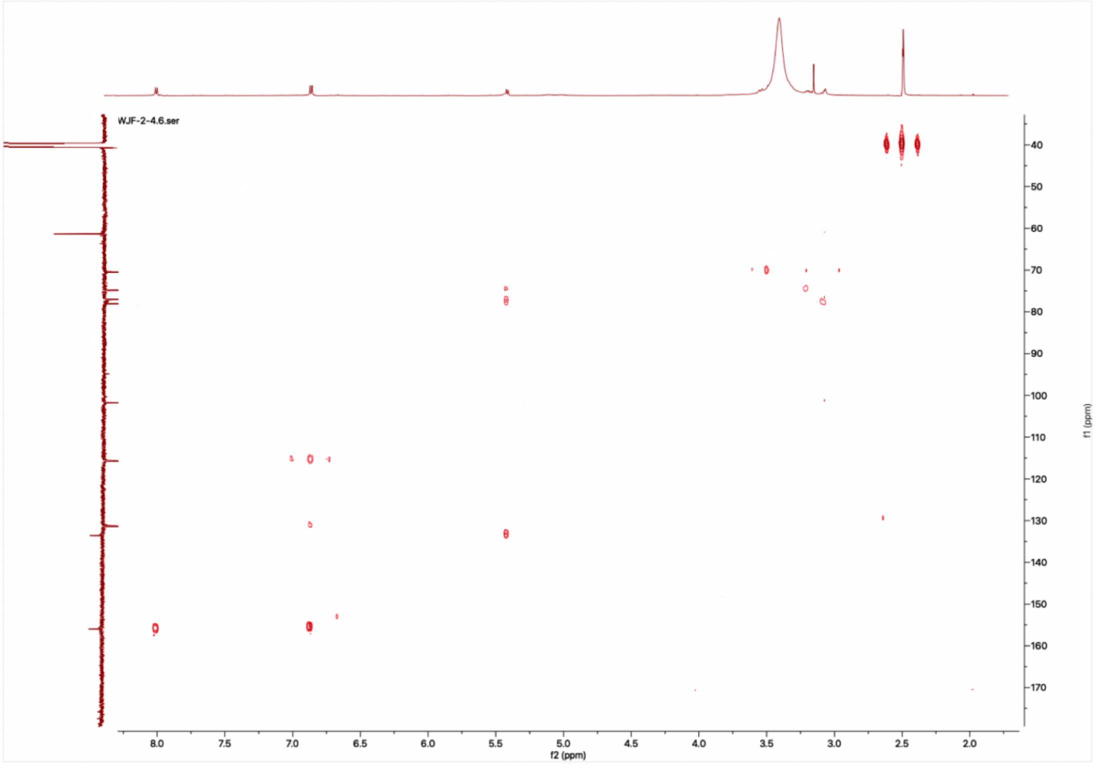

Figure S5. HMBC spectrum of compound **1**

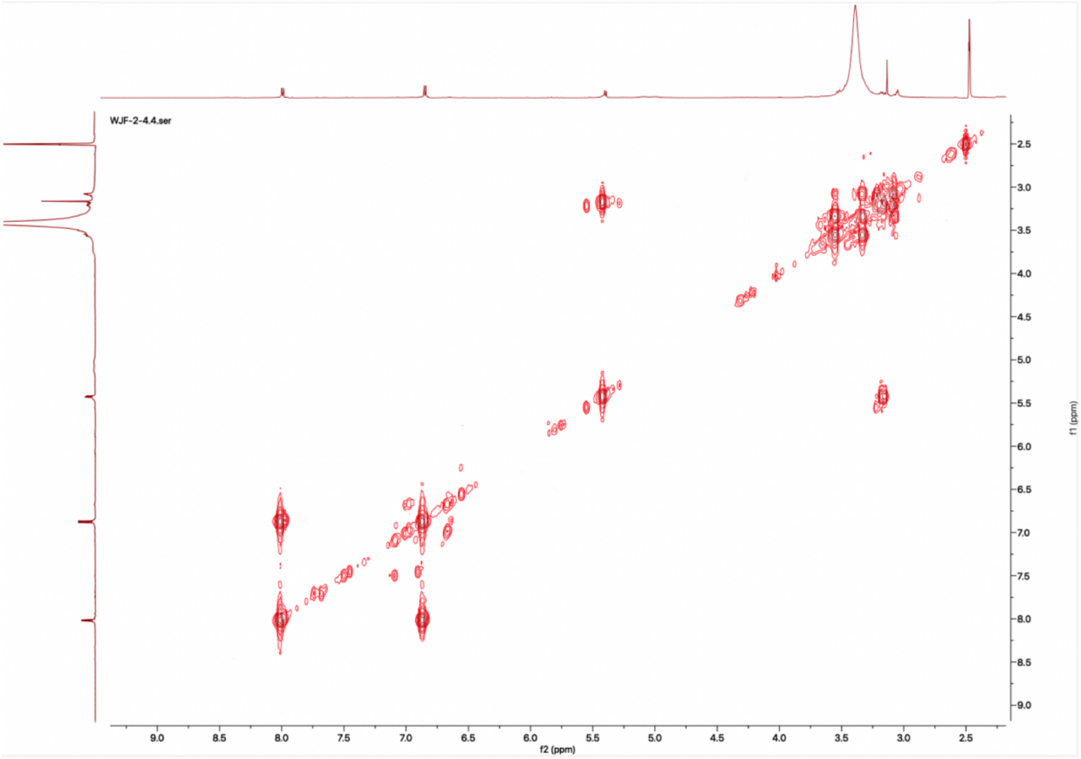

Figure S6. COSY spectrum of compound **1**

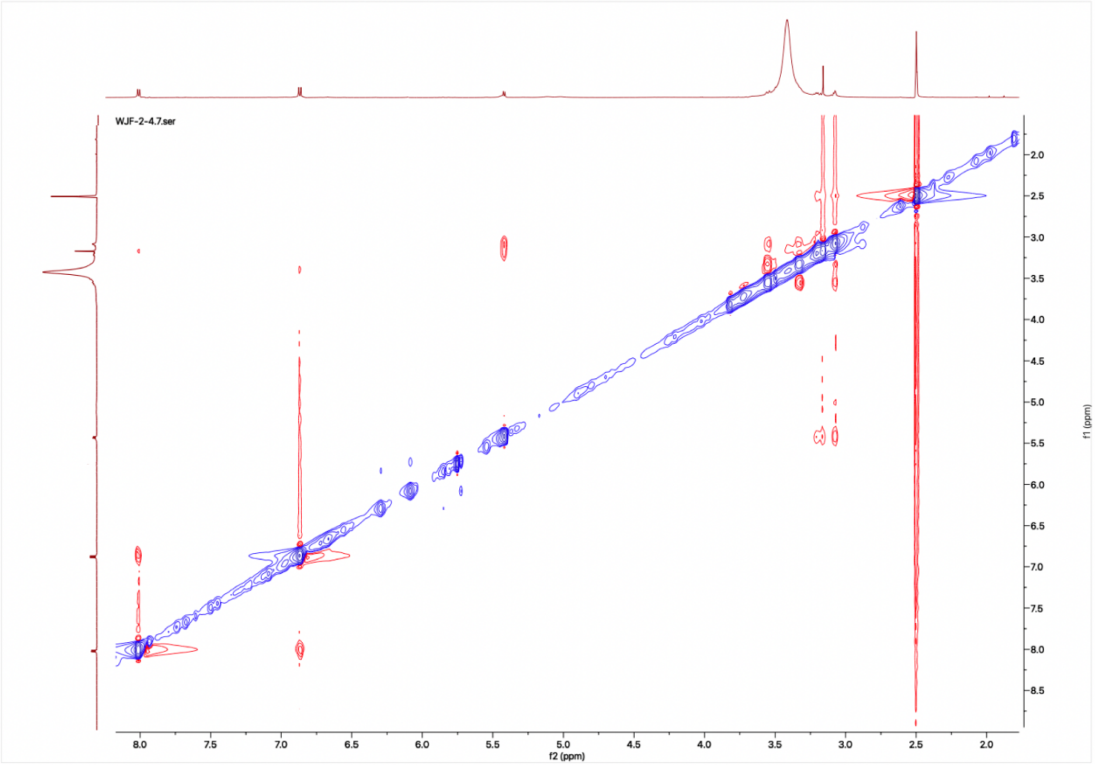

Figure S7. NOESY spectrum of compound **1**

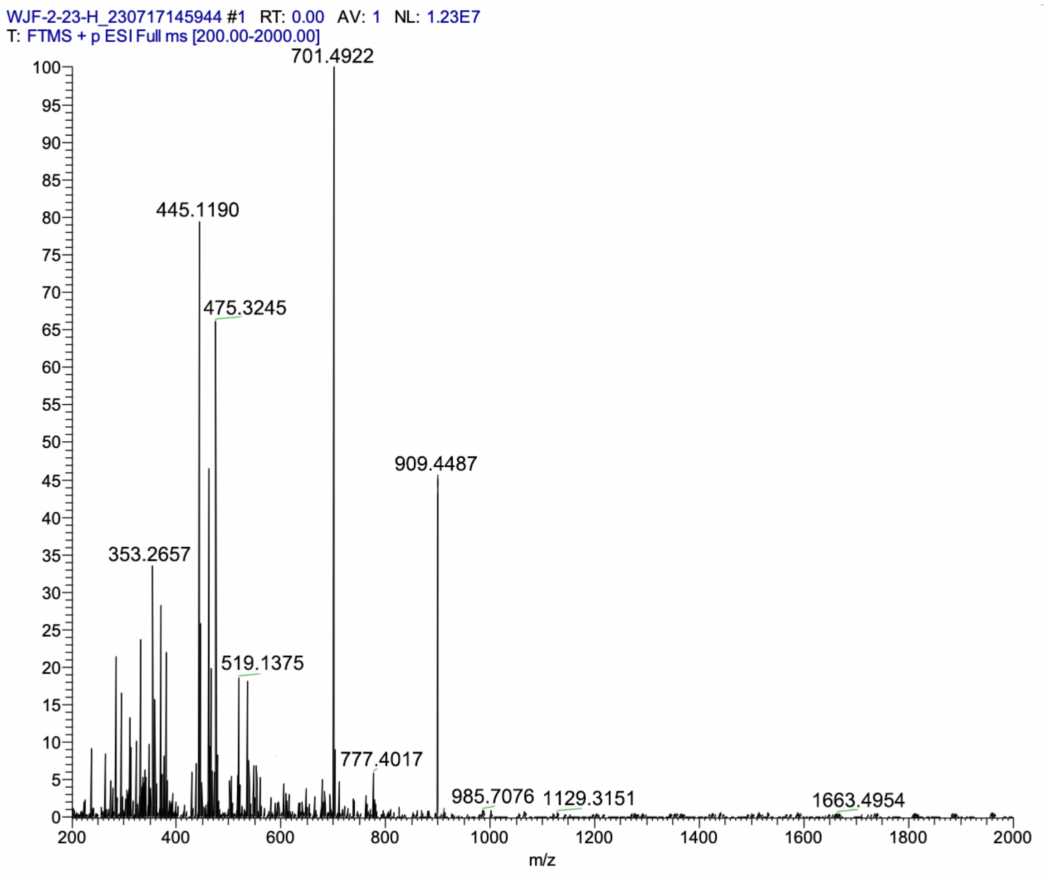

Figure S8. HR-ESI-MS spectrum of compound **2**

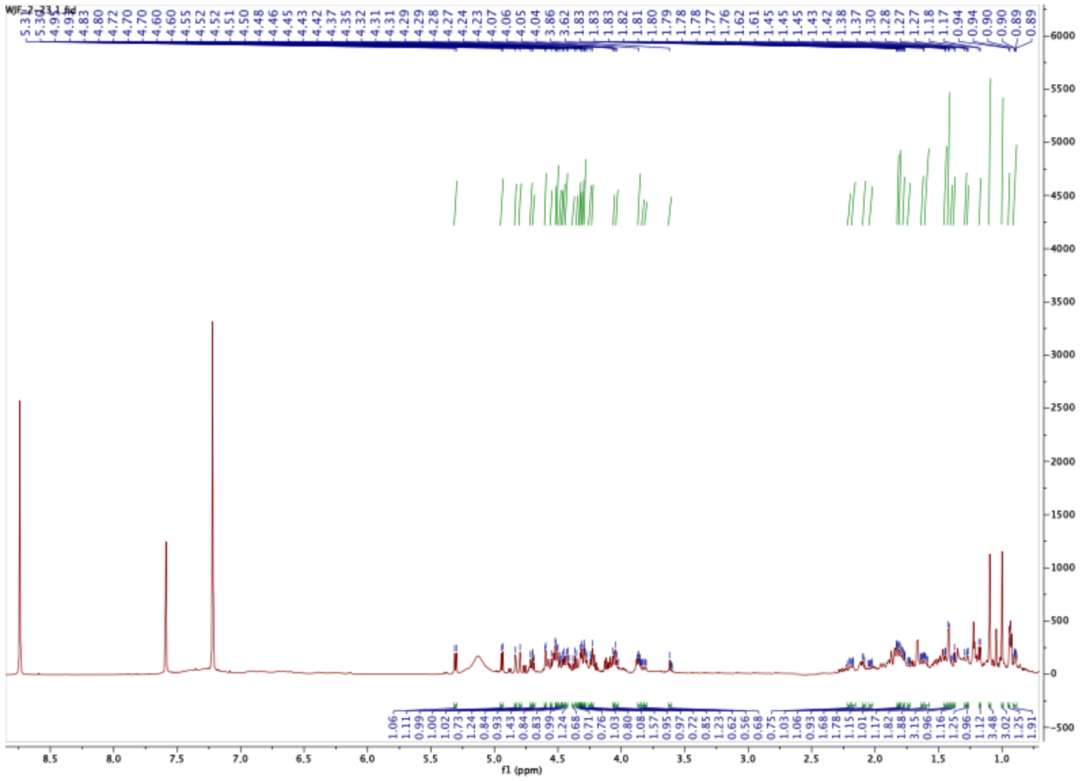

Figure S9. ^{1}H-NMR spectrum of compound **2** (in Pyridine-*d*$_5$, 600 MHz)

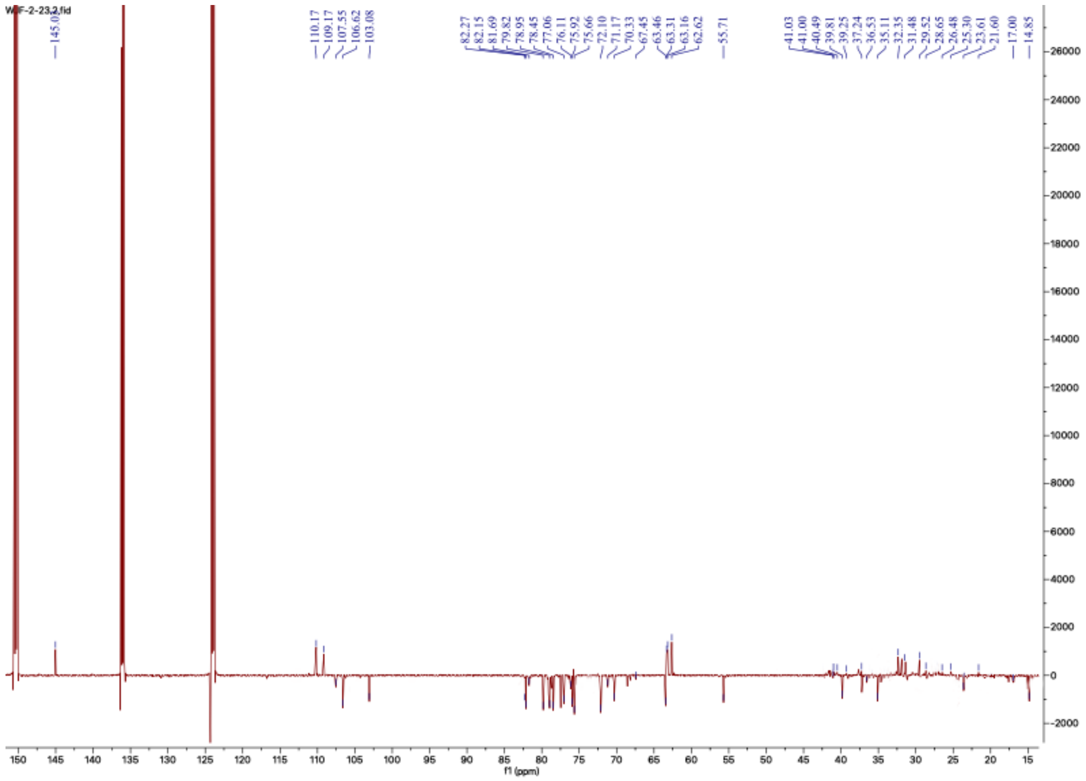

Figure S10. ^{13}C-NMR-APT spectrum of compound **2** (in Pyridine-*d*$_5$, 150 MHz)

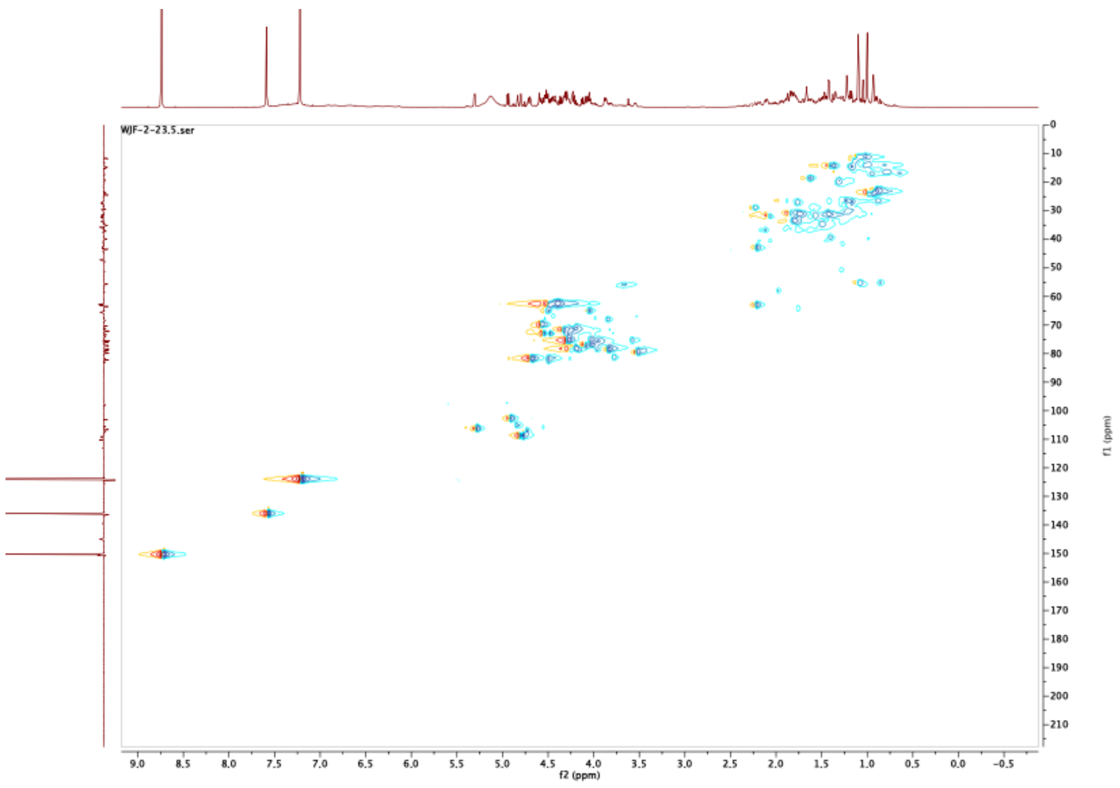

Figure S11. HMQC spectrum of compound **2**

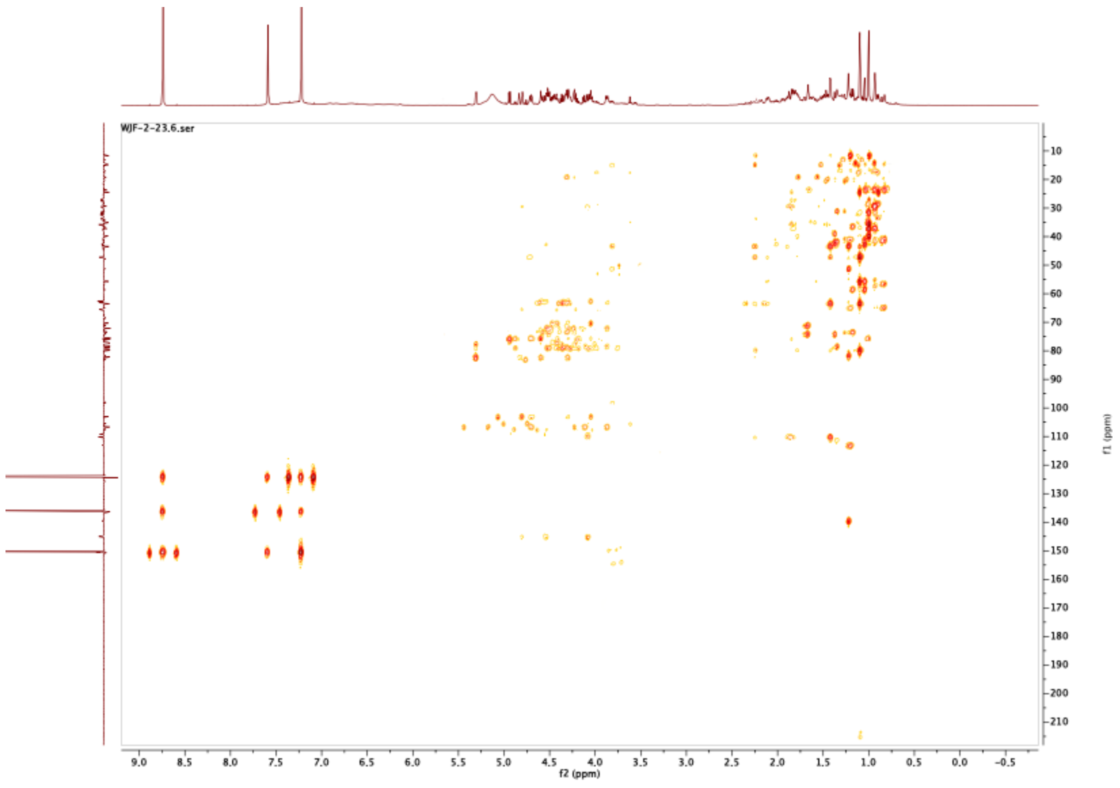

Figure S12. HMBC spectrum of compound **2**

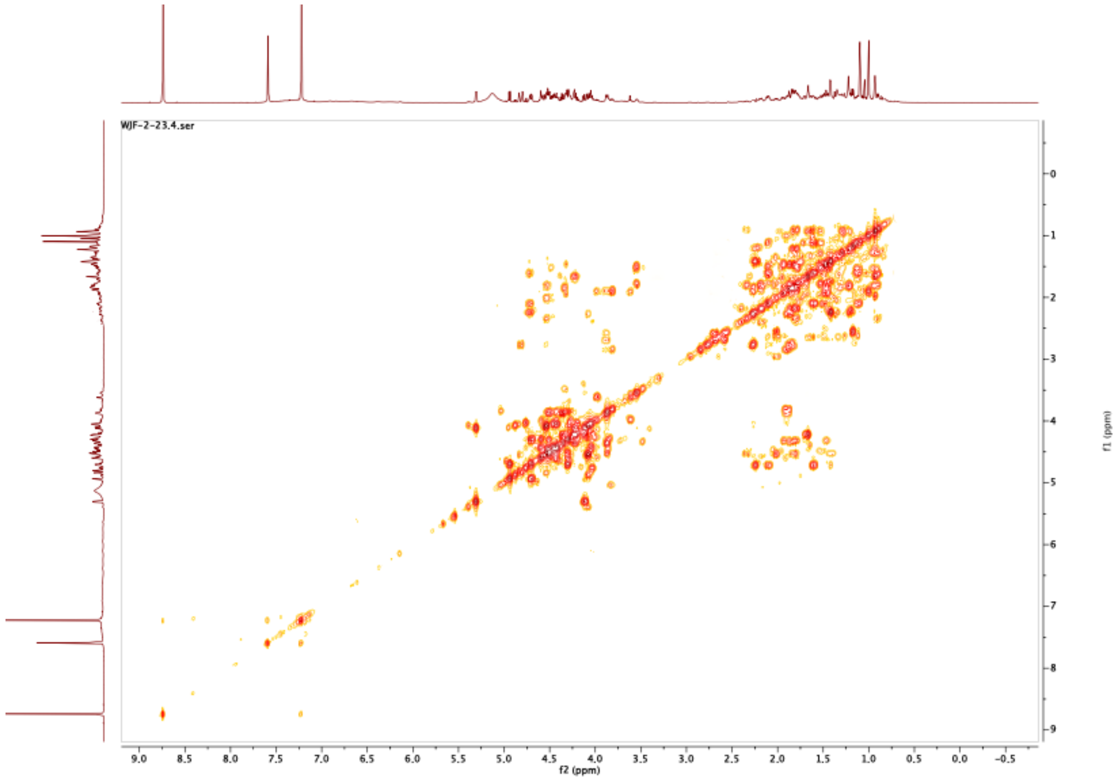

Figure S13. COSY spectrum of compound **2**

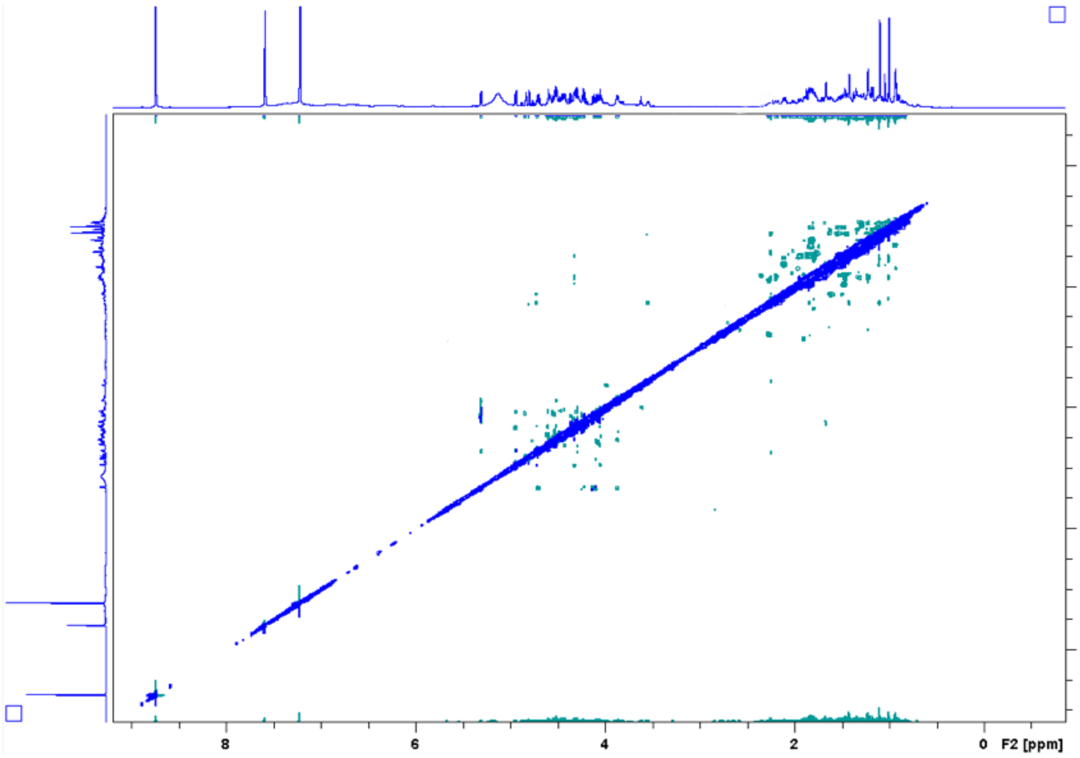

Figure S14. NOESY spectrum of compound **2**
